# Editorial: Public health innovations inspired by transgender day of visibility

**DOI:** 10.3389/fpubh.2026.1837175

**Published:** 2026-04-09

**Authors:** Kemesha Gabbidon, Katie Heiden-Rootes, Olivia T. Van Gerwen

**Affiliations:** 1 Department of Psychology, University of South Florida, St. Petersburg, FL, United States; 2 School of Medicine, Family and Community Medicine, St. Louis University, St Louis, MO, United States; 3 Division of Infectious Diseases, University of Alabama at Birmingham, Birmingham, AL, United States

**Keywords:** community engaged, editorial, health disparities, systematic review, transgender health, voice intervention

It is not lost on the guest editors of this Research Topic—who all hail from U.S. states with famously anti-transgender legislative agendas (Missouri, Florida, and Alabama)—the meaningfulness of continuing to celebrate transgender visibility and actively advance health equity through this Research Topic in 2026. As researchers and clinicians who work with transgender communities, we present here nine articles that draw on global insights to inform more inclusive and effective public health and healthcare strategies that improve health outcomes for transgender people.

Dalton et al. tested the leading health disparities model for transgender people—the Gender Minority Stress Model—among transgender people of color, the majority of whom were Black/African American. The study found that intersectional stressors directly and indirectly predict depression and anxiety, underscoring how structural inequities produce multiple minority stressors that contribute to mental health disparities.

Two articles studied gender-affirming voice changes for transgender patients. Schwarz et al. highlighted the power of global resource sharing by adapting and applying a Transgender Men's Voice Questionnaire, previously validated for Portuguese transgender men, for clinical use with Brazilian transgender men undergoing masculinizing hormone therapy. The questionnaire supported clinical assessments and offered guidance on creating community support programs for issues such as pitch range, voice variability challenges, and frustration with voice modification attempts. Gahbauer et al. utilized a qualitative observational study to map the learning process of medical students in Vienna, Austria, who took part in a novel online seminar that used a voice app and self-reflection to understand gendered voice and transgender health. The authors highlighted the emotional impact of learning and developing empathy for transgender patients, a crucial aspect of training patient-centered, compassionate providers to serve the transgender community.

In their scoping review, de Souza Gonçalves et al. applied Andersen's Behavioral Model of Health Services Use to map the existing literature, characterize factors that influence access to healthcare services and goods, and identify knowledge gaps related to trans, travesti, and gender-diverse (TTGD) populations in Latin America. The review highlighted persistent structural barriers, including undertrained providers, institutional transphobia, and pathologizing protocols that hinder access to care. The authors also documented significant unmet health needs, including gender-affirming procedures, hormone management, chronic disease care, and psychological support. Finally, the review highlighted several enabling strategies- such as TTGD-led services, safe spaces, and collaborative, de-pathologized policies- that improve access and experiences of care.

Using a community-engaged online survey, Thompson et al. examined preferences for emerging long-acting HIV pre-exposure prophylaxis (PrEP) formulations relative to daily oral PrEP and explored current PrEP uptake patterns among Black and Latina transgender women in Chicago, Illinois, United States. The authors found that preferences for a monthly pill were associated with socioeconomic factors, including higher educational attainment and full-time employment, highlighting the role of structural conditions in shaping prevention choices. They also found that current PrEP users were more likely to be stably housed, while more than half did not report CDC-indicated risk factors for PrEP use, suggesting potential mismatches between existing eligibility guidelines and the lived realities of transfeminine communities.

Recognizing the critical importance of HIV PrEP but also the continued challenges of sustained uptake and adherence, Li et al. conducted a meta-analysis of 16 randomized controlled trials, evaluating the effectiveness of mobile health (mHealth) interventions in strengthening the HIV prevention cascade—from risk identification to PrEP adherence—among key populations, including men who have sex with men, bisexual individuals, sex workers, and transgender communities. The findings indicated that mHealth interventions significantly improved PrEP adherence and increased HIV testing, while also showing promise in promoting PrEP uptake. However, these interventions did not significantly reduce condomless sex during follow-up periods.

Unfortunately, trauma shapes the lives of many transgender individuals because of ingrained societal transphobia and discrimination. Experienced trauma or fear of future trauma is often a deterrent for transgender people in accessing sexual healthcare, which likely influences some of the adverse health outcomes observed in this population, such as high HIV and sexually transmitted infection prevalence. Van Gerwen et al. utilized themes from their previous qualitative work and existing validated trauma scales to develop a survey instrument intended to measure trauma among transgender women. Of the 105 transgender women surveyed, high rates of experienced trauma were reported in multiple domains (such as sexual/relationship trauma, healthcare-related trauma, crime-related trauma, discrimination, and gender dysphoria). The initial psychometric evaluation of the survey was promising, giving it the potential to facilitate trauma assessment in sexual healthcare settings and provide trauma-informed care for transgender women.

The mainstays of masculinizing gender-affirming hormone testosterone therapy are weekly injections or daily gel application. While both routes can be effective, they do not suit all patients, especially those seeking longer-acting options in an era of uncertain access to gender-affirming care services. Hudda et al. conducted a retrospective chart review of 13 individuals on long-acting, subcutaneously implanted testosterone pellets, which are dosed every 3–6 months. The researchers found their sample to have therapeutic serum testosterone levels with an acceptable safety profile. These results demonstrated the possibility of long-acting testosterone formulations to provide patient-centered adherence solutions.

Improving the delivery of gender-affirming care services expands beyond the clinic walls. Pharmacists are an integral part of the healthcare system, with frequent patient contact and communication. In their grounded theory study based in Brazil, de Souza Gonçalves et al. conducted interviews with 10 pharmacists and found that structural and educational reforms, along with an emphasis on narratives of transgender lived experiences, would facilitate their capacity to care for this population and thus, better meet their unique needs.

Collectively, these studies reveal that improving transgender health is inseparable from confronting the conditions that produce inequities. Achieving equitable care requires sustained attention to intersectionality, investment in provider training, and the development of flexible, community-informed, and rights-based approaches that center transgender leadership. This Research Topic highlights the power of global and interdisciplinary collaboration in addressing the complex social determinants that shape transgender health and affirms that meaningful progress depends on centering the voices, expertise, and lived realities and needs of transgender people from diverse backgrounds.

